# *De novo* transcriptome assembly for rudimentary leaves in *Litchi chinesis* Sonn. and identification of differentially expressed genes in response to reactive oxygen species

**DOI:** 10.1186/1471-2164-15-805

**Published:** 2014-09-20

**Authors:** Xingyu Lu, Hyeji Kim, Silin Zhong, Houbin Chen, Zhiqun Hu, Biyan Zhou

**Affiliations:** College of Horticulture, South China Agricultural University, Guangzhou, 510642 China; Department of Horticulture and Landscape Architecture, Purdue University, West Lafayette, IN 47907-201 USA; State Key Laboratory of Agrobiotechnology, School of Life Sciences, Chinese University of Hong Kong, Shatin, Hong Kong

**Keywords:** Rudimentary leaf, Abortion, Flowering, Transcriptome, Reactive oxygen species, Litchi

## Abstract

**Background:**

Litchi is an evergreen woody tree widely cultivated in subtropical and tropical regions. Defective flowering is a major challenge for litchi production in time of climate change and global warming. Previous studies have shown that high temperature conditions encourage the growth of rudimentary leaves in panicles and suppress litchi flowering, while reactive oxygen species (ROS) generated by methyl viologen dichloride hydrate (MV) promote flowering and abortion of rudimentary leaves. To understand the molecular function of the ROS-induced abortion of rudimentary leaves in litchi, we sequenced and *de novo* assembled the litchi transcriptome.

**Results:**

Our assembly encompassed 82,036 unigenes with a mean size of 710 bp, and over 58% (47,596) of unigenes showed significant similarities to known sequences in GenBank non-redundant (nr) protein database. 5,865 unigenes were found to be differentially expressed between ROS-treated and un-treated rudimentary leaves, and genes encoding signaling components of plant hormones such as ABA and ethylene were significantly enriched.

**Conclusion:**

Our transcriptome data represents the comprehensive collection of expressed sequence tags (ESTs) of litchi leaves, which is a vital resource for future studies on the genomics of litchi and other closely related species. The identified differentially expressed genes also provided potential candidates for functional analysis of genes involved in litchi flowering underlying the control of rudimentary leaves in the panicles.

**Electronic supplementary material:**

The online version of this article (doi:10.1186/1471-2164-15-805) contains supplementary material, which is available to authorized users.

## Background

Litchi is one of the most important subtropical evergreen fruit trees in southern Asia. A major factor determining litchi crop production is the competition between vegetative and reproductive growth during floral differentiation. Floral initiation in litchi could be triggered by low temperatures and enhanced by droughts in autumn and winter [1, 2]. In the following spring, the apical buds will break and elongate as the air temperature and soil moisture rise. Next, the axillary or apical panicle primordia will emerge and become visible as “whitish millets” [3]. At this millet stage, floral buds are considered to be mixed buds containing axillary or apical panicle primordia, leaf primordia and rudimentary leaves. Whether these mixed buds could develop into flowers are largely dependent on the environmental conditions. Under normal climate conditions, the growth of panicle primordia will prevail and the rudimentary leaves will abscise. However, if the buds are exposed to high temperature, the rudimentary leaves could develop into fully expanded leaves and the panicle primordia will cease to develop and shrink [4]. Suppressing the growth of the rudimentary leaves encourages panicle development. Warm winter and hot spring potentially resulted from global warming present a major threat to litchi flowering. In order to develop a counter measure, it is important to understand the genetics behind litchi bud development, and the abortion of the rudimentary leaves.

We have previously shown that ethylene could promote abortion of rudimentary leaves. It is associated with an increase in H_2_O_2_ [5], a type of reactive oxygen species (ROS). It is well known that environmental stresses stimulate ROS production in plants [6]. They act as key signals in response to stresses [7]. In *Arabidopsis*, H_2_O_2_ has been shown to be elevated in leaves at the time of floral transition [8]. It has been proposed that H_2_O_2_ bursts generate a signal in leaves associated with either the induction of flowering or leaf senescence [9]. The ethylene-induced H_2_O_2_ might act as a signal which induces abortion of rudimentary leaves in the panicle and promotes flowering in litchi. Methyl viologen dichloride hydrate (MV) is a ROS producer in plants. It can generate superoxide by accepting an electron from PSI to become a reduced free radical, which is immediately reoxidized by dioxygen, producing superoxide in chloroplast [10]. MV also induces the increase of superoxide production in mitochondria, where complexes I and III are the major electron donors [11]. Superoxide is then transformed to H_2_O_2_ by superoxide dismutase [12, 13]. When the litchi leafy panicles were treated with MV, it was found that ROS accumulated, the rudimentary leaves abscised and the numbers of flowers per panicle increased [14]. We have also shown that ROS increased the expression of *LcLFY* and *LcAP1* in panicles [14, 15]. Studies on *Arabidopsis* and other plants indicated that LFY (LEAFY) is a transcription factor which determines the floral meristem identity and is strongly expressed in the flower buds [16, 17]. Constitutive expression of *Arabidopsis AP1* (*APETALA1*) has also been shown to promote flowering in citrus [18]. *AP1* is involved in the transition from floral induction to flower formation and constitutes a hub in the corresponding network of regulatory genes [19, 20]. Beside a few ROS responsive EST clones derived from a suppression subtractive hybridization (SSH) library screen [21], little is known about the transcriptional network controlling litchi flowering.

Without a litchi reference genome, *de novo* transcriptome assembly using Illumina short RNA-Seq reads is the most cost effective approach for generating a large collection of ESTs suitable for subsequent transcriptome analysis. This method has been successfully applied to Chinese bayberry (*Myrica rubra*) and watermelon (*Citrullus lanatus* (Thunb.) Matsum*. & Nakai* var. *lanatus*) for fruit development and ripening studies [22, 23], pear (*Pyrus pyrifolia*) for bud dormancy analysis [24, 25], and litchi (*Litchi chinesis*) and melon (*Cucumis melo*) for fruit abscission study [26, 27]. Though litchi fruit transcriptome sequencing data were published [27], those of leaves are unknown. In this study, we have constructed a litchi reference transcriptome for rudimentary leaves using Illumina RNA Sequencing (RNA-seq). We also used digital gene expression assay to profile the transcriptome dynamics of ROS treated rudimentary leaves, in order to elucidate genetic network of the ROS-induced abortion.

## Results

### MV induced abortion of rudimentary leaves

**Figure 1:**
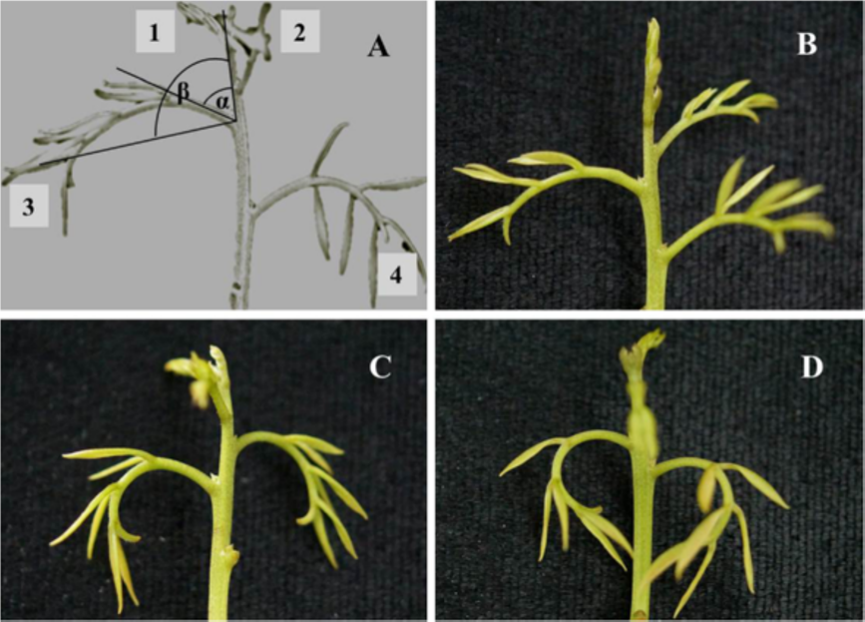
**Images of the new flushes. (A)** Image of a new flush showing the first to forth rudimentary leaves, **(B)** Image of a new flush in 0 h of ROS treatment, **(C)** Image of a new flush in 5 h of ROS treatment, **(D)** Image of a new flush in 10 h of ROS treatment. α, proximal angle of the rudimentary leaves; β, distal angle of the rudimentary leaves; numbers from 1 to 4 indicate the first, the second, the third and the fourth rudimentary leaves respectively.

In our previous study, we found that an early sign of abortion of rudimentary leaves was downward growth of the leaves [4]. To confirm the effect of MV treatment, shoot cuttings were treated with water or MV in a growth chamber. The proximal angle (α) and the distal angle (β) of the third and the fourth MV-treated rudimentary leaves were measured (Figure 1A). We used this experimental system for the easy control of light intensity and temperature. Furthermore, in our preliminary study, it was confirmed that detached shoots could survive in water without wilting for at least 2 d. To avoid the immediate effect of the cutting from trees, treatments were carried out after the shoots were placed in water for 2 h. The results showed that proximal angle α had no significant change during the treatments, while the distal angle β significantly increased after the treatment (Table 1). The ROS-treated rudimentary leaves showed epinasty as characterized by downward curvature of leaves (Figure 1B, C, D), presenting an early sign of abortion [4].

**Table 1:** **Effects of ROS on angles of the rudimentary leaves**

Time of treatment	Proximal angle α (°)	Distal angle β (°)
0 h	51.03 ± 1.09 a	83.60 ± 1.79 c
5 h	51.83 ± 1.03 a	111.36 ± 2.16 b
10 h	52.88 ± 1.01 a	126.02 ± 2.16 a

Values are means ± SE from 30 rudimentary leaves. The differences among all the treatment means were evaluated by Duncan’s multiple range tests at a 0.01 probability level using a SPSS program (SPSS Inc. Chicago, IL, USA). Different lower-case letters indicate significant differences.

### Sequence, assembly and annotation of a litchi reference transcriptome

**Figure 2:**
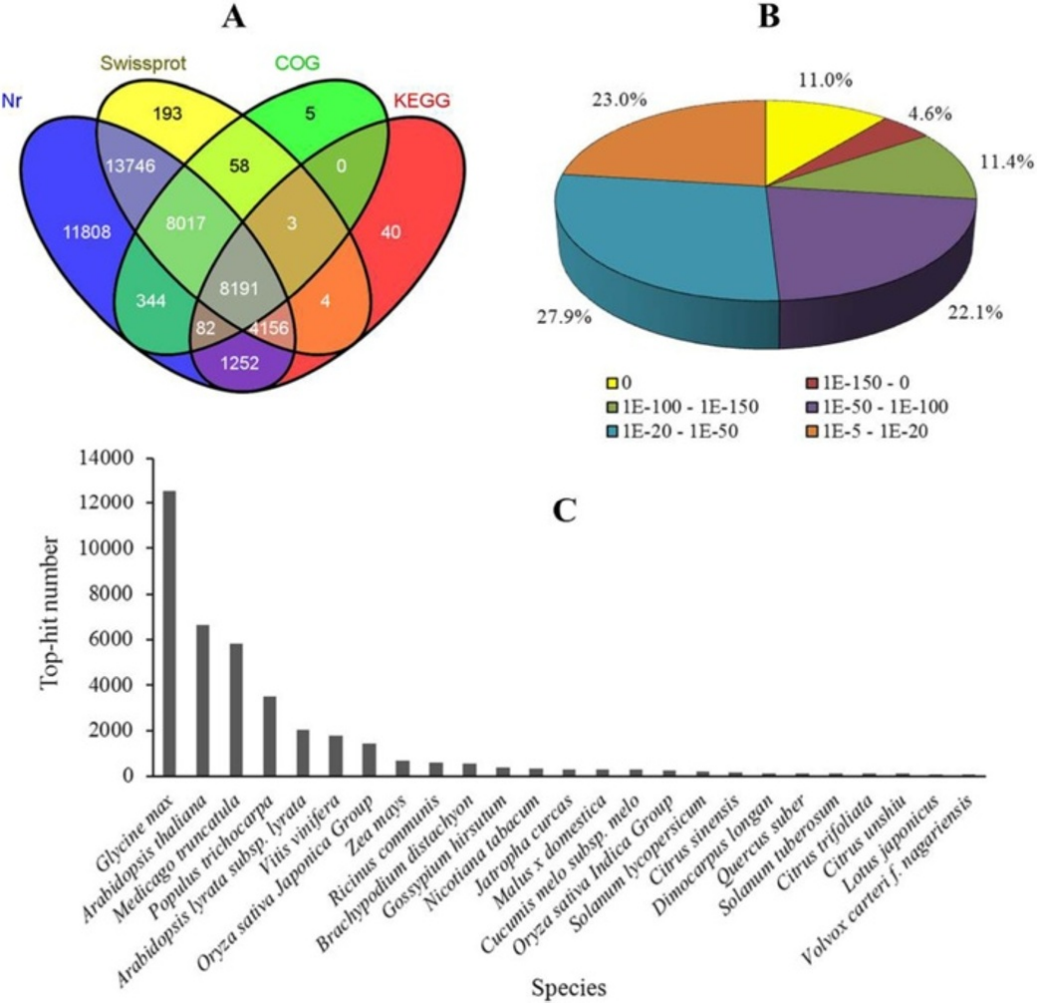
**Characteristics of homology search of litchi unigenes. (A)** Venn diagram of number of unigenes annotated by BLASTx with a cut-off E-value 1e^−05^ against protein databases. Numbers in the circles indicate the number of unigenes annotated by single or multiple databases, **(B)** E-value distribution of the top BLASTx hits against the nr database, **(C)** Number of unigenes matching the 25 top species using BLASTx in the nr database.

To obtain a reference litchi transcriptome for the rudimentary leaves, a RNA-Seq library has been constructed using RNA from all leaf samples. As shown in Table 2, we have generated 6.13 G of total nucleotides with a Q20 percentage of 97.8%. The Trinity package assembled 82,036 unigenes with a mean size of 710 bp (Table 3). The size distributions of these unigenes are shown in Additional file 1. 58% (47,596/82,036) of unigenes could be annotated by BLASTx (E-value < 1e^−5^) using the NCBI nr database, while 34,368 were annotated using the Swiss-Prot protein database. In addition, 13,728 and 16,700 unigenes could be annotated according to the Kyoto Encyclopedia of Genes and Genomes (KEGG) and Cluster of Orthologous Groups of protein (COG) database, respectively. About 10% (8,191/82,036) unigenes could be assigned to a homolog in all four databases (Figure 2A). Based on the NCBI nr database, 23.0% of the unigenes showed homology (1e^−20^ < E-value < 1e^--5^), 50.0% of those showed strong homology (1e^−100^ < E-value < 1e^−20^) and the remaining 27.0% were very strong homology (E-value < 1e^−100^) to available plant sequences (Figure 2B). As shown in Figure 2C, 28,556 unigenes were annotated to 4 top-hit species, including *Glycine max*, *Arabidopsis thaliana*, *Medicago truncatula* and *Populus trichocarpa*. 23,278 unigenes could be classified into 3 gene ontology (GO) categories: cellular component, biological process, and molecular function (Additional file 2). 13,728 unigenes of the rudimentary leaves in litchi were mapped into 274 KEGG pathways (Additional file 3). The maps with the highest unigene representation were ribosome pathway (ko03010) with 629 ungenes counted, followed by protein processing in endoplasmic reticulum (ko04141), starch and sucrose metabolism (ko00500), RNA transport (ko03013), purine metabolism (ko00230), Spliceosome (ko03040) and plant hormone signal transduction pathway (ko04075).

**Table 2:** **Throughput and quality of RNA-seq of the reference library and the DGE libraries**

Libaries	Total	Total Nucleotides	Q20 percentage	N percentage	GC percentage
	Reads	(nt)			
Reference library	61,338,190	6,133,819,000	97.82%	0.01%	46.73%
Lc0h-1	30,382,747	1,488,754,603	98.51%	0.01%	46.11%
Lc0h-2	30,181,112	1,478,874,488	98.53%	0.01%	45.85%
Lc5h-1	28,942,560	1,418,185,440	98. 45%	0.01%	46.11%
Lc5h-2	32,276,093	1,581,528,557	98.48%	0.01%	46.28%
Lc10h-1	30,733,719	1,505,952,231	98.50%	0.01%	45.90%
Lc10h-2	31,382,314	1,537,733,386	98.51%	0.01%	45.83%

One reference library was constructed by mixing RNA extracted from ROS-treated rudimentary leaves in 0 h, 5 h and 10 h of treatment. 6 DGE libraries were constructed from 0 h, 5 h and 10 h of ROS-treated rudimentary leaves. Each time point of treatment had 2 biological replicates. All libraries were sequenced using HiSeq 2000. Q20 percentage indicates the percentage of sequences with sequencing error rate lower than 1%. N percentage is the percentage of nucleotides which could not be sequenced.

**Table 3:** **Summary of the transcriptome assembly**

Assembly statistics	
Total number of unigenes	82, 036
Mean length of unigenes (bp)	710
Sequences with E-value < 1e^−5^ against nr	47, 596

### Identify differentially expressed genes using digital gene expression tag (DGE)

To identify differentially expressed genes in response to ROS, 6 DGE libraries have been generated from ROS-treated rudimentary leaves at 0 h, 5 h and 10 h post treatment, with two biological replicates. The libraries produced over 1.41 G of 49 nt single-end read data with a Q20 percentage of about 98%. The percentage of unassigned base “N” is 0.01% and the average GC content is around 45% (Table 2). To assay the normality of the RNA-Seq data in the 6 DGE libraries, we calculated the distribution of unique reads in each DGE libraries. This value is the ratio of the number of bases in a gene covered by unique mapping reads to the total bases in that from our transcriptome reference database. The distribution over different reads abundance categories showed similar patterns among all six libraries. Above 36% of the sequences have coverage more than 80% (Additional file 4). Next we calculated the unigene expression using the uniquely mapped DGE reads and normalized the results to RPKM. Results from the two biological replicates are highly similar, suggesting good reproducibility of the method (Additional file 5). We performed a pairwise comparison using 0 h as the control, and 5 h, or 10 h as the treatments. We also identified differentially expressed unigenes with FDR (false discovery rate) ≤ 0.001 and absolute value of log_2_ Ratio ≥ 1. As a result, 5,865 unigenes were referred to as differentially expressed genes (DEGs) and used for the subsequent analysis (Additional file 6).

### GO-term analysis of differentially expressed genes

**Figure 3:**
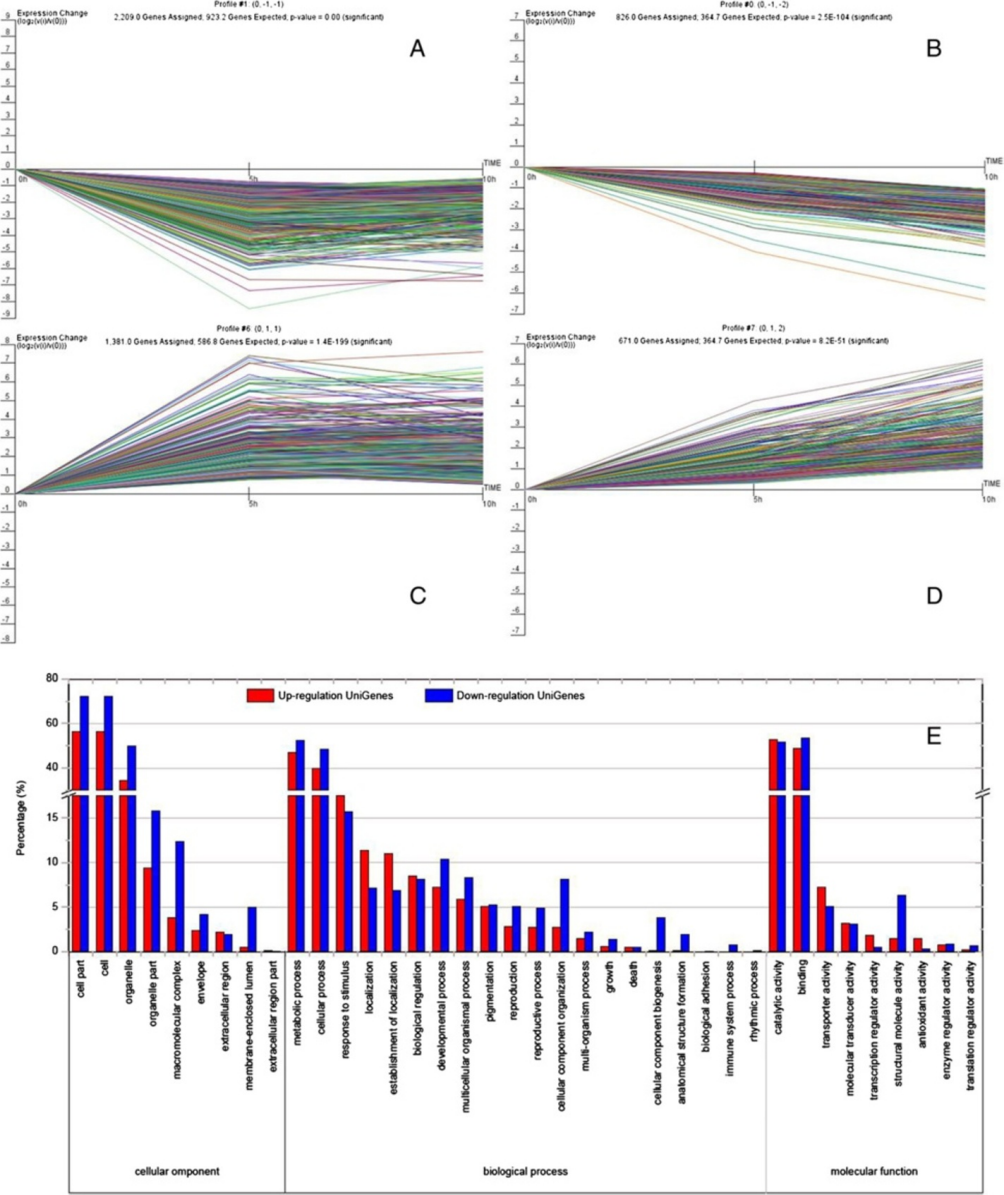
**DEGs expression profiles (A-D) and their GO classification (E) in the MV-generated ROS-treated rudimentary leaves.** Profile 1 **(A)** and profile 0 **(B)** indicating a down-regulated trend, profile 6 **(C)** and profile 7 **(D)** indicating an up-regulated trend during 0 to 10 h of ROS treatment. The down-regulation DEGs are union of DEGs in profile 1 and 0. The up-regulation DEGs are union of DEGs in profile 6 and 7.

To examine the expression profile of the 5,865 DEGs, the expression data υ (from 0 h to 10 h of ROS treatment) were normalized to 0, log_2_ (υ_5h_/υ_0h_), log_2_ (υ_10_/υ_0h_). 5,623 DEGs could be clustered into 8 profiles by Short Time-series Expression Miner software (STEM), in which 5,087 were clustered into 4 profiles (p-value ≤ 0.05), including two down-regulated patterns (Profile 1 and Profile 0) and two up-regulated patterns (Profile 6 and Profile 7) (Figure 3 A-D, Additional file 7). Profile 1 and 0 contained 2,209 and 826 DEGs respectively, while Profile 6 and 7 contained 1,381 and 671 DEGs (Additional file 7). Next, the DEGs within the up- and down-regulated cluster groups were subjected to GO-term analysis. They were classified into 3 main categories including cellular component, biological process, and molecular function. Under the cellular component category, a large number of up-regulation, as well as down-regulation DEGs were categorized as cell part, cell and organelle. Under biological process category, most of those were classified into cellular process and metabolic process. For molecular function category, catalytic activity and binding were the top abundant subcategories (Figure 3 E).

### KEGG pathway enrichment analysis of differentially expressed genes

DEGs were subjected to KEGG pathway enrichment analysis. 27.4% (1,606/5,865) of the DEGs could be annotated. The 10 top KEGG pathways with the highest representation of the DEGs are shown in Table 4. The ribosome (ko03010), plant hormone signal transduction (ko04075), glycolysis/gluconeogenesis (ko00010), starch and sucrose metabolism (ko00500), purine metabolism (ko00230), phenylpropanoid biosynthesis (ko00940), pyrimidine metabolism (ko00240), pyruvate metabolism (ko00620), DNA replication (ko03030) and plant-pathogen interaction (ko04626) pathways are significantly enriched. The 122 unigenes among 837 DEGs (14.58%) in profile 1, and 11 unigenes accounting for 5.16% of 213 in profile 0 were annotated to ribosome pathway, whereas in profile 6 and 7, only 2 unigenes accounting for 0.65% of 307 DEGs, 7 accounting for 7.64% of 144 DEGs were annotated to this pathway.

**Table 4:** **10 top KEGG pathways with high representation of the DEGs**

Pathways	No. of DEGs with pathway annotation					Pathway ID
	All profiles	Profile 1	Profile 0	Profile 6	Profile 7	
	(% of 1606)	(% of 837)	(% of 213)	(% of 307)	(% of 144)	
Ribosome	147 (9.15%)	122 (14.58%)	11 (5.16%)	2 (0.65%)	11 (7.64%)	ko03010
Plant hormone signal transduction	65 (4.05%)	17 (2.03%)	9 (4.23%)	22 (7.12%)	9 (6.25%)	ko04075
Glycolysis/Gluconeogenesis	62 (3.86%)	32 (3.82%)	7 (3.29%)	12 (3.90%)	10 (6.94%)	ko00010
Starch and sucrose metabolism	57 (3.55%)	20 (2.39%)	14 (6.57%)	11 (3.58%)	8 (5.56%)	ko00500
Purine metabolism	47 (2.93%)	32 (3.82%)	10 (4.69%)	4 (1.30%)	1 (0.69%)	ko00230
Phenylpropanoid biosynthesis	44 (2.74%)	12 (1.37%)	6 (2.82%)	12 (3.91%)	6 (4.17%)	ko00940
Pyrimidine metabolism	44 (2.74%)	30 (3.58%)	11 (5.16%)	2 (0.65%)	1 (0.69%)	ko00240
Pyruvate metabolism	43 (2.68%)	20 (2.39%)	3 (1.41%)	12 (3.91%)	7 (4.86%)	ko00620
DNA replication	40 (2.49%)	31 (3.70%)	9 (4.22%)	0 (0.00%)	0 (0.00%)	ko03030
Plant-pathogen interaction	35(2.18%)	16 (1.91%)	7 (3.29%)	6 (1.95%)	1 (0.69%)	ko04626

### DEGs in the signal transduction pathways in response to ROS

Table 5 shows the number of the DEGs involved in the plant hormone signal transduction pathway during 0 to 10 h of ROS-treatment. Numbers of those DEGs in the 4 significantly different expression patterns were calculated. A total of 57 DEGs were annotated in the plant hormone signal transduction pathways, including auxin, cytokinine, gibberellin, abscisic acid, ethylene, brassinosteroid and jasmonic acid.

**Table 5:** **Number of DEGs involved in the plant hormone signal transduction pathway during 0 to 10 h of ROS treatment**

Components	All profiles	Profile 1	Profile 0	Profile 6	Profile 7
Auxin					
*AUX1*	4	0	1	2	1
*AUX/IAA*	1	1	0	0	0
*GH3*	7	4	1	0	0
*SAUR*	3	0	0	0	0
Cytokinine					
*CRE1*	2	0	2	0	0
*AHP*	1	0	0	1	0
*B-ARR*	1	0	0	1	0
*A-ARR*	1	0	1	0	0
Gibberellin					
*3GID1*	2	0	0	1	1
*DELLA*	2	1	0	1	0
Abscisic acid					
*PYR/PYL*	3	0	1	2	0
*PP2C*	2	0	0	0	2
*SnRK2*	4	0	0	1	3
*ABF*	3	0	0	1	2
Ethylene					
*ETR*	3	0	0	3	0
*CTR1*	1	0	0	1	0
*MPK6*	2	2	0	0	0
*EBF1/2*	2	0	0	2	0
*EIN3*	3	0	0	3	0
*ERF1/2*	2	0	0	2	0
Brassinosteroid					
*BKI1*	1	0	0	0	0
*BSK*	1	0	1	0	0
*TCH4*	4	3	0	1	0
*CYCD3*	2	0	2	0	0
Jasmonic acid					
*JAR1*	3	2	0	0	0
*JAZ*	5	4	0	0	0

**Figure 4:**
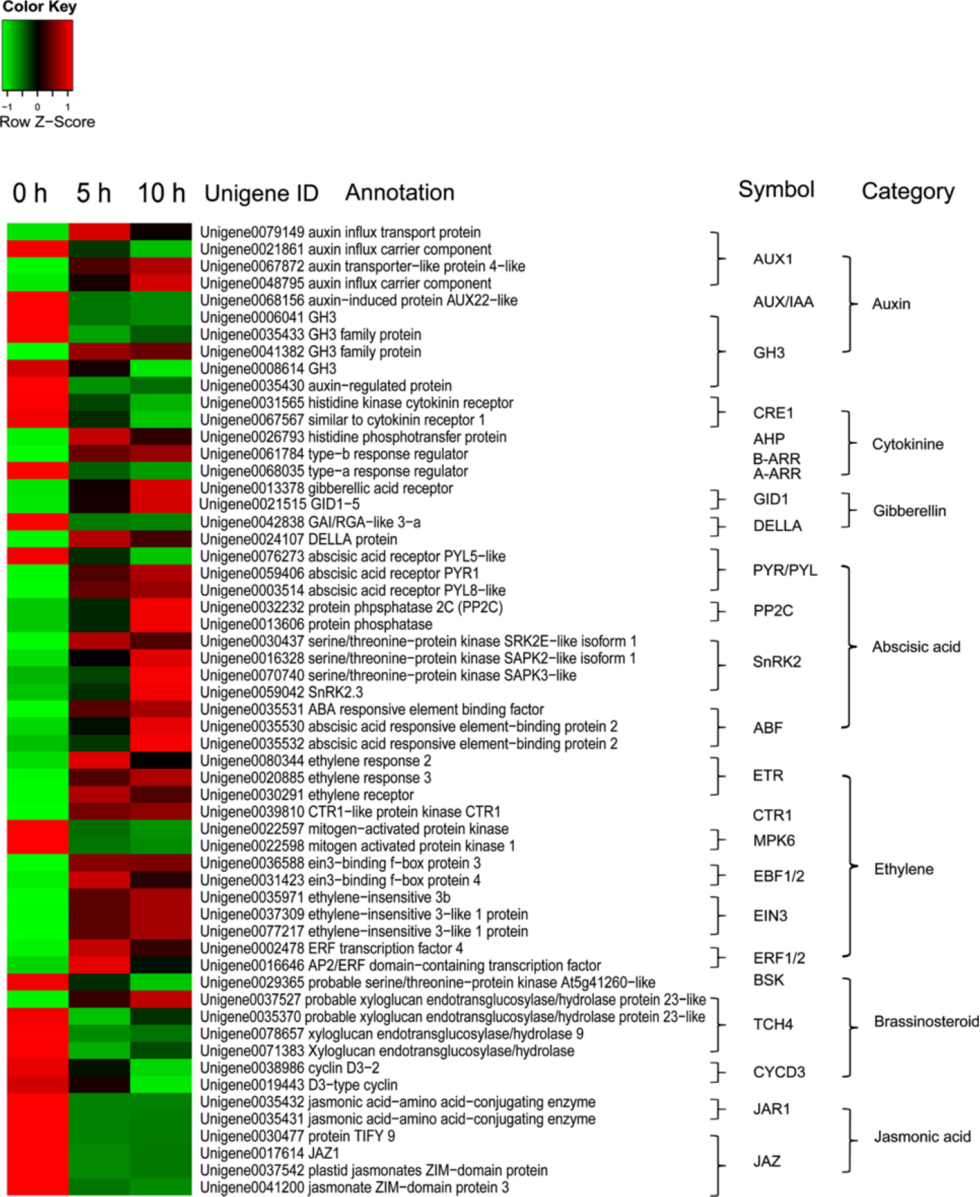
**Heat map diagram of expression levels for DEGs annotated in the plant hormone signal transduction pathways analyzed by KEGG.** Data for gene expression level were normalized to z-score. The original KEGG map is shown in Additional file 9.

In the auxin signal transduction pathway, 4 unigenes encoding auxin influx transport protein (AUX1) were found to be differentially expressed, among which two were annotated to profile 6, and one to profile 7 showing different pattern of up-regulation. It was found that 7 DEGs encode gretchenhagen-3 (GH3) or GH3 family protein. Four of them were clustered to profile 1, and 1 to profile 0 showing down-regulated trends. Only 1 DEG belonging to profile 1 was annotated to auxin-induced protein AUX/IAA. In the auxin signal transduction pathway, 7 out of the 15 DEGs showed down-regulated trends, and 3 of those showed up-regulated trends, indicating that DEGs of the down-regulated trends in this pathway were more than those of up-regulated trends. Similar results were found in the cytokinine, gibberellin, brassinosteroid and jasmonic acid signal transduction pathway. In the cytokinine signaling pathway, 2 unigenes encoding cytokinin receptor 1B or 1 (CRE1) and 1 encoding type-a response regulator (A-ARR) showed down-regulated trends, while the Unigene0026793 encoding histidine phosphotransfer protein (AHP) showed an up-regulated trend. In the gibberellin signal transduction pathway, 4 unigenes encoding gibberellic acid receptor or DELLA protein were found to be differentially expressed, where 1 was identified as a down-regulated profile, and the other as up-regulated profiles. In the brassinosteroid signaling pathway, 6 out of the 8 DEGs showed down-regulated trends, while 1 showed as an up-regulated trend (Table 5, Figure 4).

In the abscisic acid signal transduction pathway, 11 out of the 12 DEGs were clustered to profile 6 or 7 showing up-regulated trends. They encode abscisic acid receptors PYR (PYRabactin resistance)/PYL (PYR1-like), type 2C protein phosphatase (PP2C), SNF1 related protein kinase 2 (SnRK2), and ABA responsive element binding factor (ABF). Only Unigene0076273 encoding abscisic acid receptor showed a down-regulated trend. In the ethylene signal pathway, 11 out of 13 DEGs were also clustered to up-regulated profiles, including 3 unigenes encoding ethylene receptor (ETR), 2 encoding ein3-binding F-box protein (EBF1/2), 3 encoding ethylene-insensitive 3b (EIN3) and 2 encoding ERF transcription factor ERF1/2. Only 2 unigenes (Unigene0022597 and Unigene0022598) encoding mitogen activated protein kinase (MPK6) showed down-regulated trends (Table 5, Figure 4). These results showed that DEGs of the up-regulated trend in the abscisic acid and ethylene signaling pathway were much more than those of down-regulated trend, suggesting that most genes involved in these hormone signal transduction pathway were induced by ROS.

### Confirm unigenes expression using real-time quantitative reverse transcription PCR

**Figure 5:**
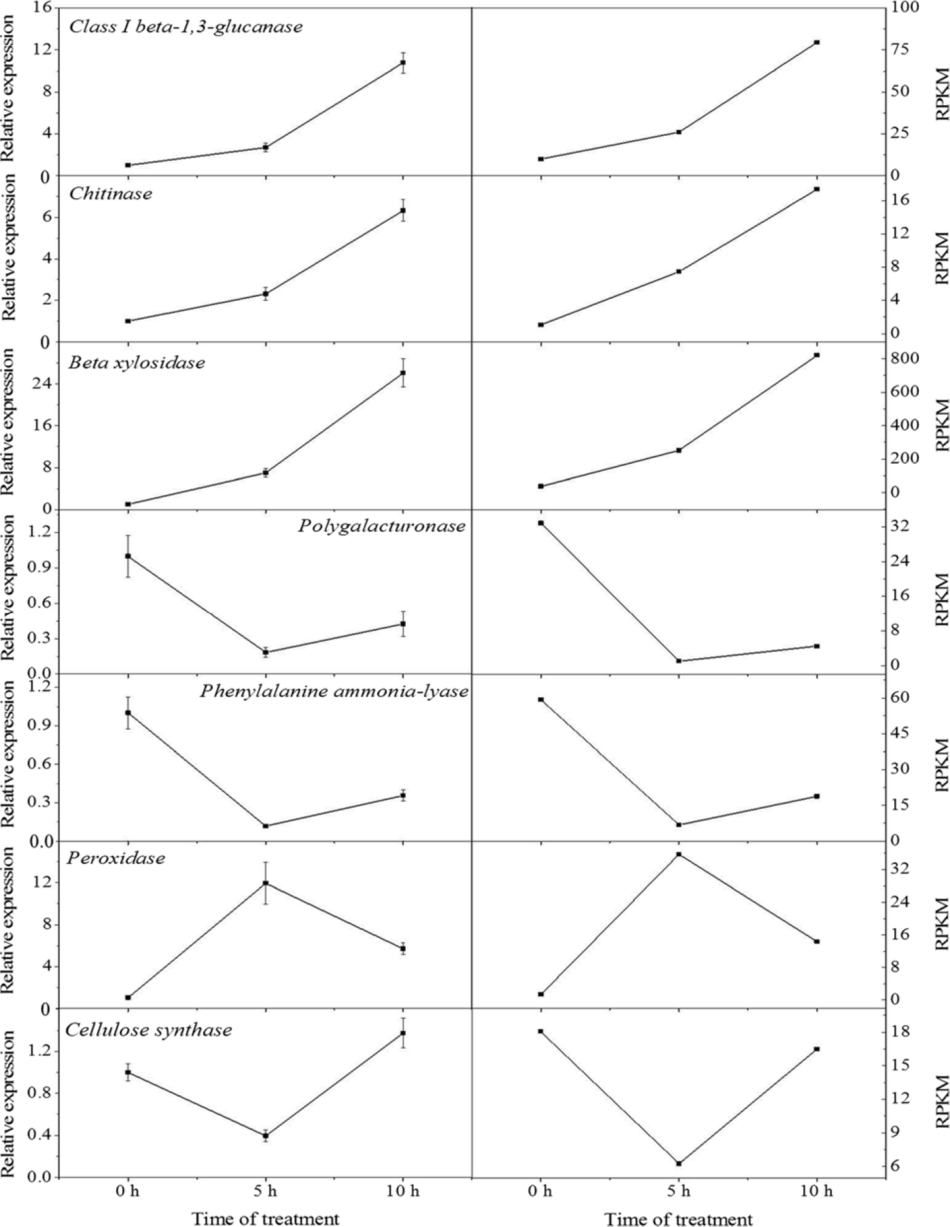
**Candidate unigene expression levels revealed by qRT-PCR (left side) and RNA-seq (right side).** Data from qRT-PCR are means of three replicates and bars represent SE. RPKM from RNA-seq are means of two replicates.

**Figure 6:**
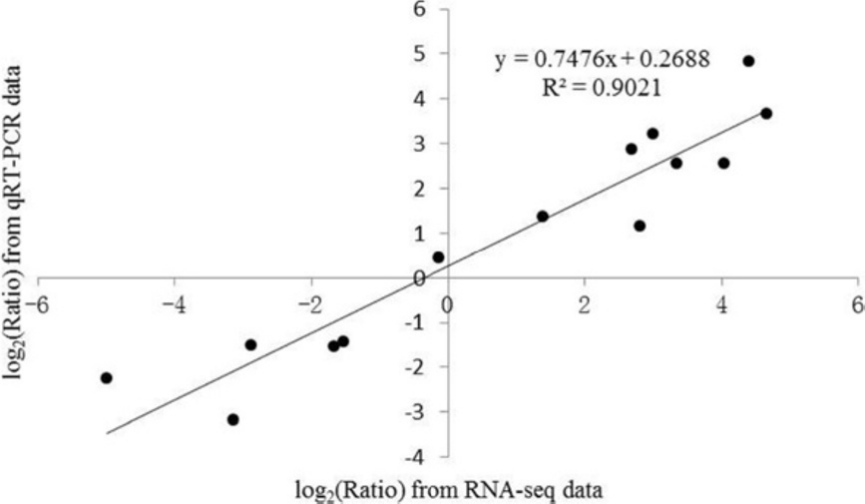
**Coefficient analysis of fold change data between qRT-PCR and RNA-seq. Seven unigenes were selected for qRT-PCR.** Data indicating relative transcript level from qRT-PCR are means of three replicates, and RPKMs from RNA-seq are means of two replicates. Scatterplots were generated by the log_2_ expression ratios from RNA-seq (x-axis) and qRT-PCR (y-axis).

To confirm the accuracy and reproducibility of the transcriptome analysis results, 7 unigenes were selected for real-time quantitative reverse transcription PCR (qRT-PCR) validation. RNA samples from the ROS-treated rudimentary leaves were used as templates. Primers of the candidate unigenes are shown in Additional file 8. The expression profiles of the candidate unigenes revealed by qRT-PCR data were consistent with those derived from sequencing (Figure 5). Linear regression analysis of the fold change of the gene expression ratios between RNA-seq and qRT-PCR showed significantly positive correlation (Figure 6), confirming our transcriptome analysis.

### Expression levels of the candidate genes in response to ROS

**Figure 7:**
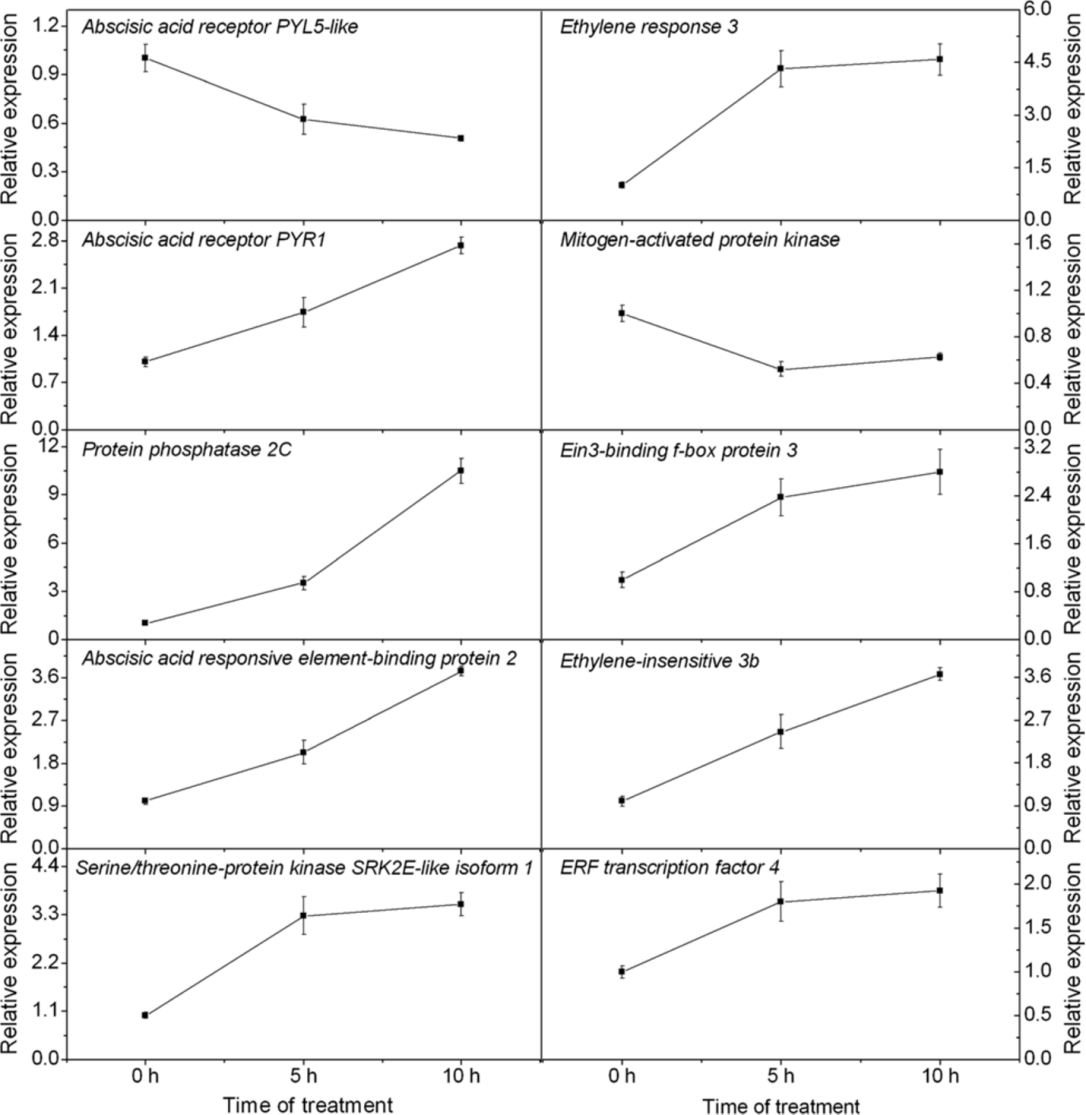
**Expression levels of the candidate unigene encoding the ABA (left side) and ethylene (right side) signal transduction components revealed by qRT-PCR.** Data from qRT-PCR are means of three replicates and bars represent SE.

Based on the KEGG pathway enrichment analysis, it was found that most of the unigenes encoding the ABA and ethylene signal transduction components were induced by ROS. We then selected 10 candidate unigenes encoding these components and analyzed their expression levels during 0 to 10 h of ROS treatment. Primers of the candidate unigenes are shown in Additional file 8. The ABA and ethylene signal transduction components are shown in Additional file 9. Analysis of the gene expression revealed by qRT-PCR shows that genes encoding the ABA signal transduction components, including the abscisic acid receptor PYR1, protein phosphatase 2C, ABA responsive element-binding protein 2 and serine/threonine-protein kinase SRK2E-like isoform 1 increased while that of the PYL5-like decreased during 0 to 10 h of treatment. Genes encoding the ethylene signal transduction components, including the ethylene response 3, Ein3-binding f-box protein 3, ethylene-insensive 3b and ERF transcription factor 4 increased while that of the mitogen-activated protein kinase decreased by the treatment (Figure 7).

## Discussion

Stresses induce flowering in evergreen woody fruit trees [28–30]. ROS act as stress signals and are recognized as important signaling components in a wide range of processes. They are generated in chloroplast and peroxisomes in the light, in mitochondria in the dark and in non-green tissues [31, 32]. These signals are perceived specifically by diverse mechanisms, such as the direct redox modification of transcription factors and other proteins, resulting in the regulation of transcriptome [33]. Our previous study showed that ROS, induced by MV, promoted continuative development of panicle primordia and abortion of rudimentary leaves in leafy panicle in litchi [14]. To understand the molecular mechanisms underlying flowering in litchi, identifying ROS responsive genes in rudimentary leaves is needed as well as those in panicle primordia. We had established an SSH library and had identified 93 ROS responsive genes in the panicle primordia [21]. In the present study, we sought to identify the stress-responsive genes in the litchi rudimentary leaves. To overcome the lack of a reference litchi genome, we used RNA-Seq data to *de novo* assemble a reference transcriptome. In total, our assembly contains 82,036 unigenes with a mean size of 710 bp. 47,596 unigenes were annotated to public protein databases. Using the transcriptome as a reference, we performed *DESeq* and identified 5,865 differentially expressed genes between un-treated (0 h) and ROS-treated (5 h or 10 h) rudimentary leaves. 2,052 unigenes showed up-regulated trends and 3,035 showed down-regulated trends from 0 to 10 h of treatments. Compared to the 93 ROS responsive genes identified by previous SSH experiment [21], RNA-Seq has identified significantly more DEGs in the rudimentary leaf libraries.

Plant hormones are signal molecules produced within the plant, and occur in extremely low concentrations, but regulate a wide range of processes, including determining the formation of flowers, stems, leaves, the shedding of leaves, the development and ripening of fruit, and in response to biotic and abiotic stresses. The plant hormone signals are perceived and transmitted to the nuclear by series signal transduction components to induce gene expression, resulting in a series of physiological processes. Our KEGG pathway enrichment analysis of the DEGs indicated that unigenes encoding the hormone signaling components were significantly enriched in the differentially expressed groups after MV treatment. These hormones included auxin, cytokinine, gibberellin, abscisic acid, ethylene, brassinosteroid, and jasmonic acid, suggesting that their signal components are responsive to ROS. It is believed that ROS signaling and redox balance is integrated with salicylic acid (SA) signaling [33]. SA-signaling pathway has been proved to have a role in controlling gene expression during senescence [34]. Though the SA-signaling pathway was not found to be significantly enriched in the ROS-treated rudimentary leaves, some unigenes, such as *NPR1* and *PAD4* encoding SA-signaling components were found to be differentially expressed (Additional file 6, Unigene0034828 and Unigene0014177), suggesting that SA might also be involved in the ROS-induced rudimentary leaf abortion.

Abscisic acid (ABA) is an essential hormone to control plant growth, development and adaptation to environmental stresses [35]. We found that 11 out of the 12 DEGs encoding abscisic acid signal components were up-regulated. The unigenes encoding PYR /PYL, PP2C, SnRK2, and ABF were induced by MV-driven ROS. Our gene expression levels of the components determined by qRT-PCR were consistent with those by RNA-seq, further confirming that the ABA signal transduction components were ROS responsive. ABA is essential for abscission and senescence of aged organs. It is involved in shading-induced abscission of apple fruits [36], and ethylene-associated abscission activation in citrus fruitlets [37]. In the present study, MV treatment induced downward growing of the rudimentary leaves, an early sign of abortion of the rudimentary leaves [4]. The cross-talk between ABA and ROS signaling is well known [38, 39], and ROS is also involved in the ABA-enhanced *LcAP1* expression in litchi [40]. Therefore, we hypothesize that the increase in the gene expression of ABA signaling components might play a role in the ROS-induced abortion of litchi rudimentary leaves.

We have also identified 13 differentially expressed genes encoding ethylene signaling components, and 11 of them were up-regulated after MV-treatment. Gene expression levels determined by qRT-PCR indicated that the unigenes encoding ethylene response 3, Ein3-binding f-box protein 3, ethylene-insensive 3b and ERF transcription factor 4 increased while that of the mitogen-activated protein kinase decreased by the ROS treatment, consisting with those revealed by RNA-seq. Our present study suggested that the ethylene signaling transduction components were ROS responsive. Ethylene as a gaseous hormone is involved in a variety of plant developmental adaptations including seed germination, organ senescence, fruit ripening, abscission and stress responses [41]. In agricultural practice, growers often use ethephon as ethylene producer to control rudimentary leaf growth in panicles and promote continual panicle development. We found that ethylene could increase H_2_O_2_ levels in the rudimentary leaves [5]. We also showed that the petioles of rudimentary leaf displayed downward growing after MV-ROS treatment (Figure 1), similarly to those of the ethylene-treated leaves, which is a phenomenon of epinasty, suggesting that the MV-ROS could function through ethylene to induce abscission of litchi rudimentary leaves.

In *Arabidopsis thaliana*, MV was used to study the influence of chloroplastic ROS generation at the transcriptional level [42]. We compared our DEGs in response to MV with those identified by Scarpeci et al. [42]. To our surprise, 84% (267/316) of their differentially expressed homology genes were found in our GEGs data set, including genes encoding some plant hormone signaling transduction components. Our study provided more information on the influence of MV generated-ROS on plant transcriptome.

## Conclusions

In summary, we reported a comprehensive litchi leaf EST dataset generated by *de novo* assembly of next generation sequencing data. It is a valuable resource for future litchi genomic studies and will also benefit researches in other closely related species with significant agricultural importance. The differentially expressed genes dataset will also provide useful candidate genes for functional analysis of litchi flowering underlying the control of rudimentary leaf.

## Methods

### Plant material and experiment procedures

Commercially cultivated thirteen-year-old litchi trees cv. Nuomici grafted onto ‘Huaizhi’ were selected. The trees were grown at the experimental orchard of South China Agricultural University. About 6 cm length of branches with new flushes were cut off from the trees and immediately placed in water. Two hours later, the cuttings were treated with water or solutions containing 40 μM MV (Sigma) according to the method of Zhou et al. [14]. All the cuttings were placed in a growth chamber at 160 μmol/m^2^ s^1^ photosynthetic photon flux density at 20°C. The third and the fourth of the 0 h, 5 h and 10 h MV-treated rudimentary leaves as shown in Figure 1A were sampled, frozen in liquid nitrogen and stored at −80°C for RNA-seq and qRT-PCR. The proximal angle α, and the distal angle β of the third rudimentary leaves as shown in Figure 1A were measured. The angular dimension was measured by degree.

### RNA isolation, library construction and EST sequencing

RNA of the rudimentary leaves from the 0 h, 5 h and 10 h ROS-treated rudimentary leaves were extracted using kits from Huayueyang Biotechnology Co., LTD., according to the manufacturer’s protocol. Samples were collected from replicate bundles (6–10 shoot cuttings in one bundle as one replicate). Each treatment had 2 replicates. In order to identify differentially expressed genes (DEGs) in response to ROS, RNA from the 6 samples were used to construct libraries for digital gene expression (DGE) profiling analyses. The libraries were as follows: Lc0h-1 and Lc0h-2 as replicate libraries for 0 h of ROS treatment, Lc5h-1 and Lc5h-2 for 5 h of ROS treatment, Lc10h-1 and Lc10h-2 for 10 h of ROS treatment. The transcriptome assembly library as a reference library was constructed by mixing equal amounts of RNA from the above 6 samples. Briefly, total mRNA was isolated with Oligo (dT) cellulose, fragmented and reverse transcripted with random primers. Second-strand cDNA were synthesized by DNA polymerase I and RNase H. Then the cDNA fragments were purified with QiaQuick PCR extraction kit, under gone end repair, dA-tailing and ligated to Illumina adapters. The ligation products were size fractioned by agarose gel electrophoresis, and fragments were excised for PCR amplification. The amplified fragments were sequenced using Illumina HiSeq™ 2000 by Gene Denovo Co. (Guangzhou, China).

### De novo assembly and annotation

For the assembly library, raw reads were filtered to remove those containing adapter and reads with more than 5% unknown nucleotides. Low quality reads were also remove, in which the percentage of low Q-value (≤10) base was more than 20%. Clean reads were *de novo* assembled by the Trinity Program [43]. To annotate the unigenes, we used BLASTx program (http://www.ncbi.nlm.nih.gov/BLAST/) at NCBI with an E-value threshold of 1e^−5^ to NCBI nr database (http://www.ncbi.nlm.nih.gov), the Swiss-Prot protein database (http://www.expasy.ch/sprot), the KEGG database (http://www.genome.jp/kegg), and the COG database (http://www.ncbi.nlm.nih.gov/COG). The sequence direction of the unigenes was determined according to the best alignment results. When the results were conflicted among databases, the direction was determined consecutively by nr, Swiss-Prot, KEGG and COG. When a unigene could not be aligned, sequence direction would be confirmed using ESTscan program. GO annotation was analyzed by Blast2GO software. Functional classification of the unigenes was performed using WEGO software. KEGG pathway annotation was undergone by Blastall software against the KEGG database. The dataset is available from the NCBI Short Read Archive (SRA) with an accession number SRA158542 (http://www.ncbi.nlm.nih.gov/sra).

### Expression annotation

Raw sequence data of the libraries for DGE profiling analyses were filtered to remove those containing adapter and reads with more than 10% unknown nucleotides, and reads with more than 50% of low quality base (value ≤5). Clean reads were mapped into the transcriptome reference database using SOAPaligner/soap2 software. Not more than 2 mismatch bases were permitted, and unique mapped reads were obtained. The number of unique-match reads was calculated and normalized to RPKM (reads per kb per million reads) for gene expression analysis.

Comparison of unigene expression between treatments was according to *DESeq* as described by Abders and Huber [44]. P-value corresponds to differential gene expression test. FDR is a method to determine the threshold of P-value. In this experiment, the DEGs between 0 h and 5 h, or between 0 h and 10 h of ROS treatment were restricted with FDR ≤ 0.001 and the absolute value of log_2_ Ratio ≥ 1.

Gene expression data υ (from 0 h to 10 h of ROS treatment) were normalized to 0, log_2_ (υ_5h_/υ_0h_), log_2_ (υ_10_/υ_0h_). DEGs were clustered by STEM [45]. The clustered profiles with p-value ≤ 0.05 were considered as significantly expressed. Then the DEGs were subjected to GO classifications using WEGO [46], and KEGG pathway annotation undergone by Blastall software against the KEGG database.

### Real-time PCR confirmation of the RNA-Seq data

First-strand cDNA was generated from 1 μg total RNA isolated from the rudimentary leaves using the superscript first-strand synthesis system (Invitrogen, USA). Primers for quantitative reverse transcription PCR (qRT-PCR) were designed using Primer Premier 5.0 software (Premier, Canada) and synthesized by Sangon Biotech (Shanghai) Co., Ltd. The litchi homologue *Actin* (GenBank accession number:HQ588865.1) was selected as reference. All the primers are shown in Additional file 8. qRT-PCR was performed on a Bio-Rad iQ5 Optical System Real Time PCR System (Bio-Rad, USA) using a SYBR Green based PCR assay. Each reaction mixture was 20 μL containing 6 μL of diluted first-strand cDNAs and 250 nM of each primer, SYBR Green PCR Master Mix (TaKaRa, Japan) 10 μL. The qPCRs were run as follows: 50°C for 2 min, 95°C for 10 min, followed by 40 cycles of 95°C for 30 s, 56°C for 30 s, and 72°C for 30 s in 96-well optical reaction plates (Bio-rad, USA). Each qRT-PCR analysis was performed in triplicate. Expression levels of the tested reference genes were determined by CT values and calculated by 2^-△△Ct^.

## Electronic supplementary material

Additional file 1: **Size distributions of unigenes in the reference library.** (PDF 10 KB)

Additional file 2: **GO assignment of all unigenes in the reference library.** (PDF 123 KB)

Additional file 3: **Pathway annotation of unigenes of the rudimentary leaves in litchi.** (XLSX 21 KB)

Additional file 4: **Distribution of unigenes’ coverage in the 6 DGE libraries.** (PDF 576 KB)

Additional file 5: **RPKM of the unigenes indicating gene expression levels.** A, RPKM of unigenes in two replicate libraries of Lc0h; B, RPKM of unigenes in two replicate libraries of Lc5h; C, RPKM of unigenes in two replicate libraries of Lc10h. (PDF 149 KB)

Additional file 6: **Differentially expressed unigenes.** This table shows all the DEGs and their RPKM values in the MV-treated leaves. (XLS 608 KB)

Additional file 7: **Profiles order based on the P-value significance of number assigned versus expected.** Numbers in the backets indicate the number of the DEGs assigned. (PDF 206 KB)

Additional file 8: **Primer sequences of the reference gene and candidate unigenes for qRT-PCR.** (PDF 48 KB)

Additional file 9: **Enriched plant hormone signal transduction pathway.** The signal transduction components marked with red rectangles are considered to be differentially expressed. (PDF 98 KB)

## Abbreviations

A-ARR: Type-a response regulator
ABF: ABA responsive element binding protein
AHP: Histidine phosphotransfer protein
AP1: APETALA1
AUX1: Auxin influx transport protein
AUX/IAA: Auxin-induced protein
COG: Cluster of Orthologous Groups of protein
DEG: Differentially expressed gene
DGE: Digital gene expression tag
CRE: Cytokinin receptor
GH3: Gretchenhagen-3
EBF: Ein3-binding F-box protein
EIN: Ethylene insensitive
EST: Expressed sequence tag
ETR: Ethylene receptor
GO: Gene ontology
KEGG: Kyoto Encyclopedia of Genes and Genomes
LFY: LEAFY
MPK: Mitogen activated protein kinase
MV: Methyl viologen dichloride hydrate
PYL: PYR1-like
PYR: PYRabactin resistance
qRT-PCR: Quantitative reverse transcription PCR
ROS: Reactive oxygen species
SAPK: Serine/threonine-protein kinase.
