# Supplementary figures and images for: De novo transcriptome assembly for rudimentary leaves in Litchi chinesis Sonn. and identification of differentially expressed genes in response to reactive oxygen species
Source: BMC Genomics. 2014 Sep 20;15(1):805. doi: 10.1186/1471-2164-15-805 (PMC4190417; doi:10.1186/1471-2164-15-805)

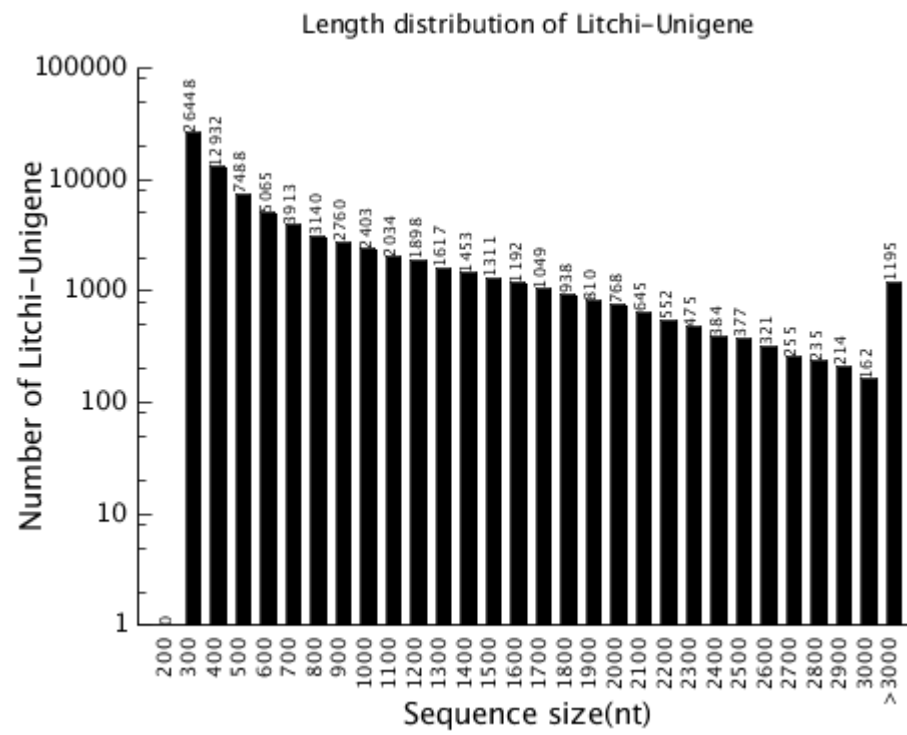

Supplement: Supplementary file 1 — Additional file 1: Size distributions of unigenes in the reference library. (PDF 10 KB) [file 12864_2014_6499_MOESM1_ESM.pdf]

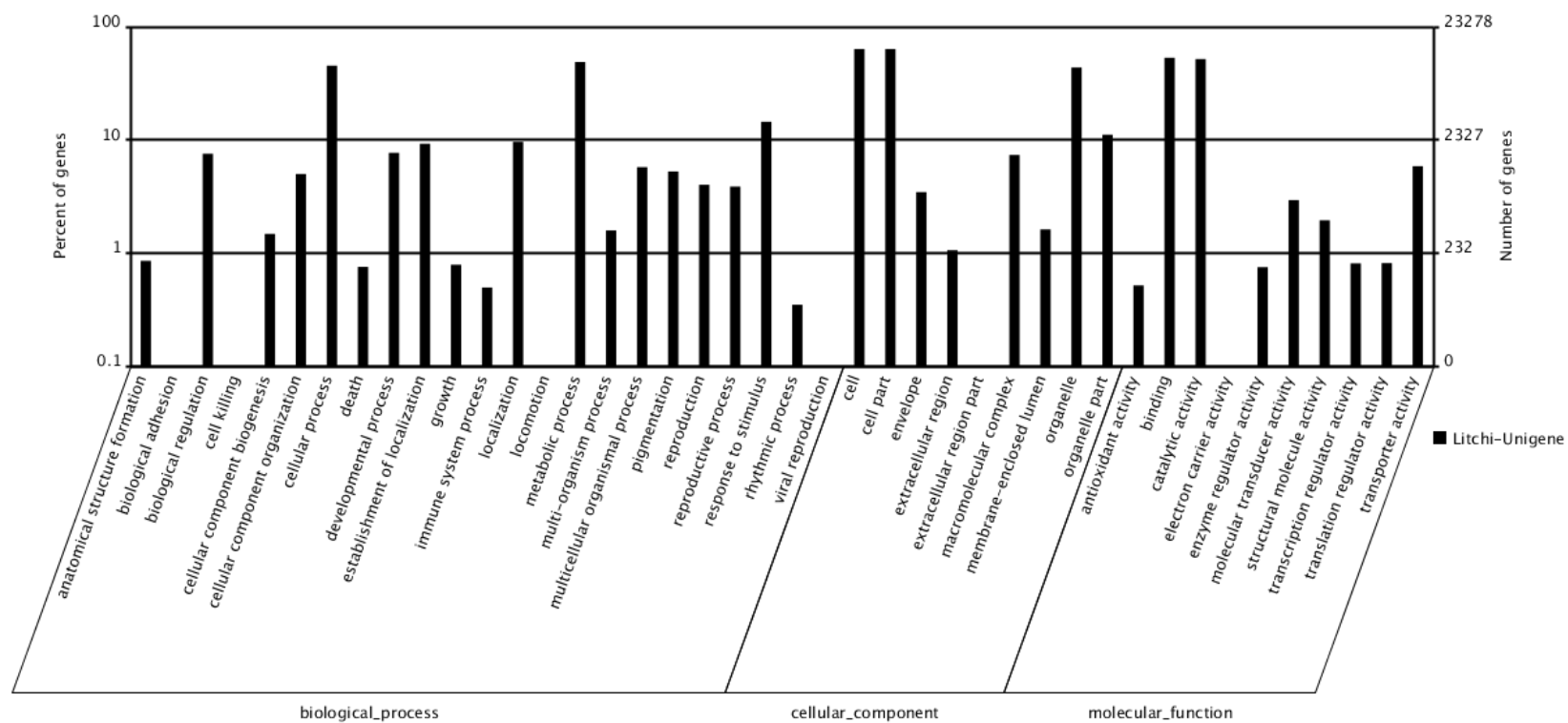

Supplement: Supplementary file 2 — Additional file 2: GO assignment of all unigenes in the reference library. (PDF 123 KB) [file 12864_2014_6499_MOESM2_ESM.pdf]

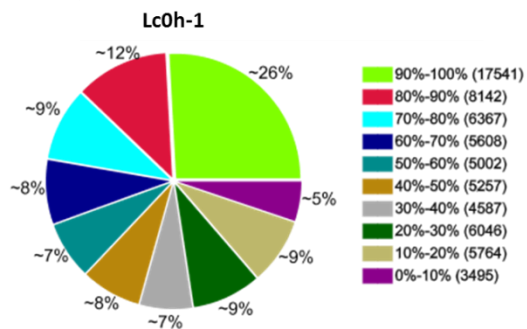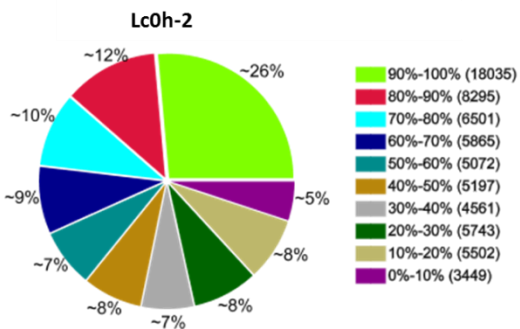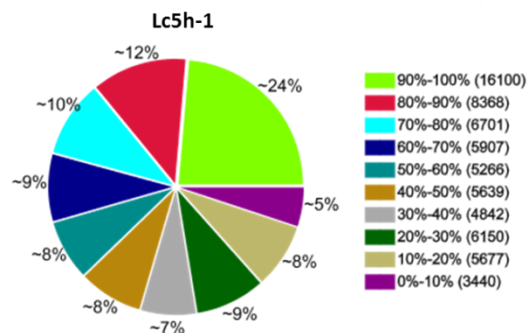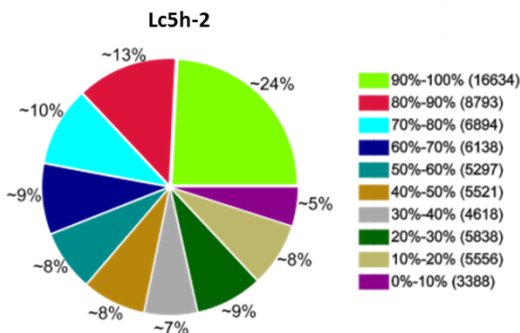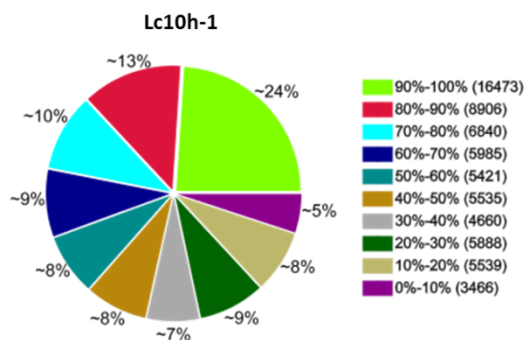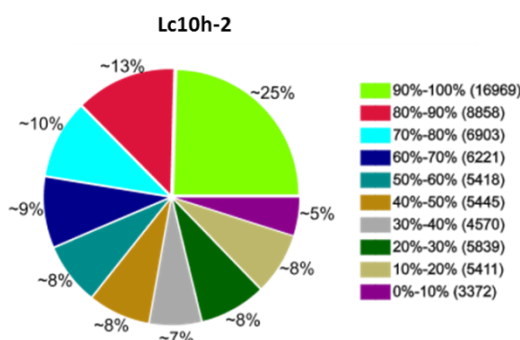

Supplement: Supplementary file 4 — Additional file 4: Distribution of unigenes’ coverage in the 6 DGE libraries. (PDF 576 KB) [file 12864_2014_6499_MOESM4_ESM.pdf]

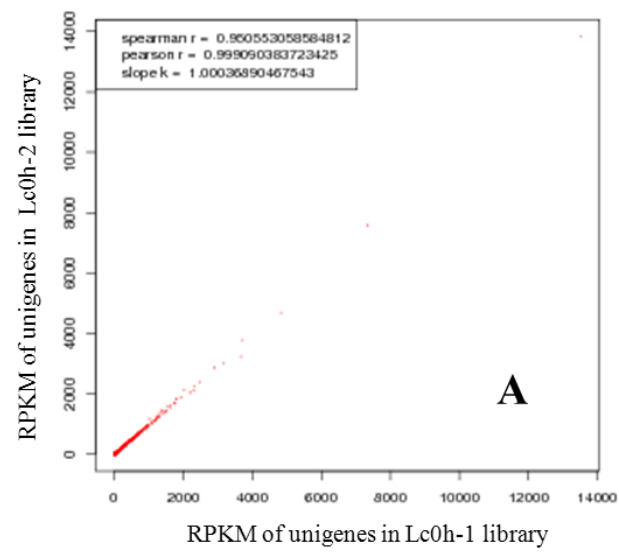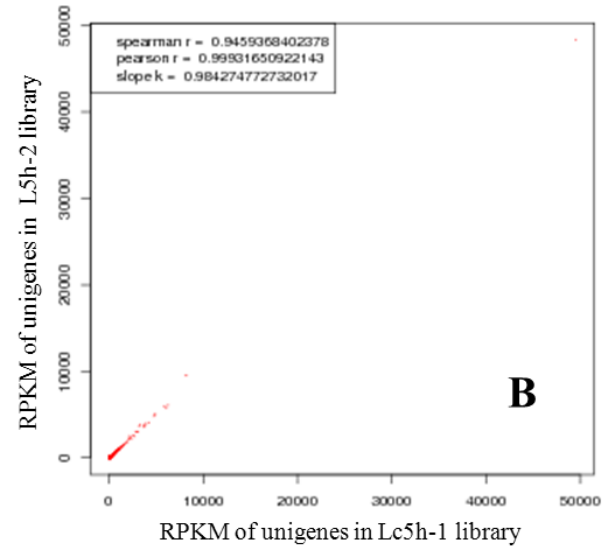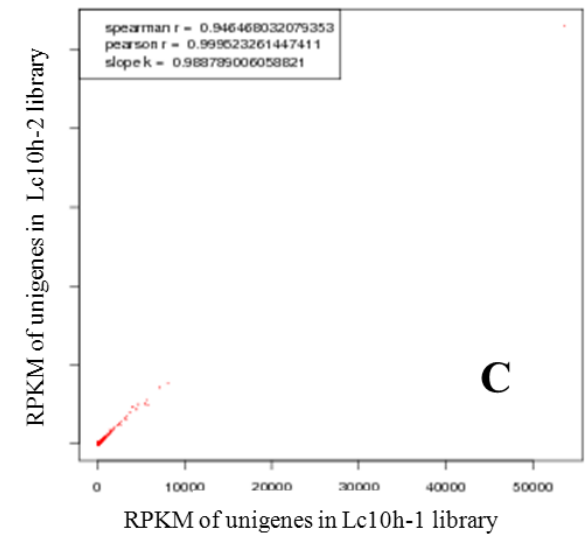

Supplement: Supplementary file 5 — Additional file 5: RPKM of the unigenes indicating gene expression levels. A, RPKM of unigenes in two replicate libraries of Lc0h; B, RPKM of unigenes in two replicate libraries of Lc5h; C, RPKM of unigenes in two replicate libraries of Lc10h. (PDF 149 KB) [file 12864_2014_6499_MOESM5_ESM.pdf]

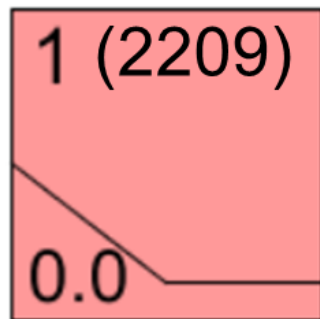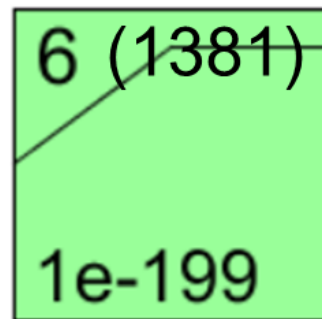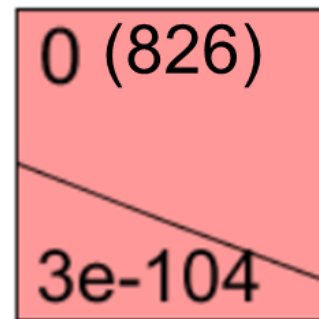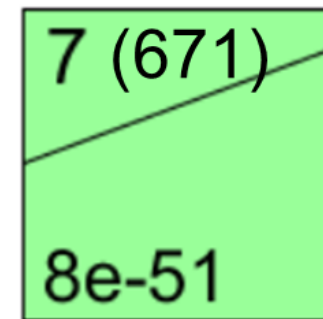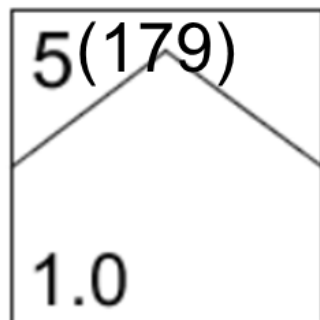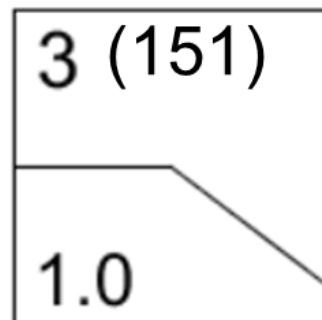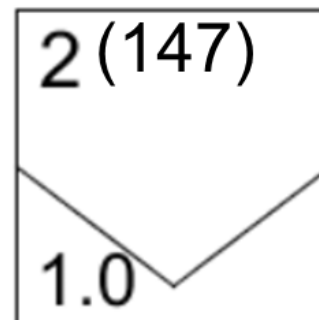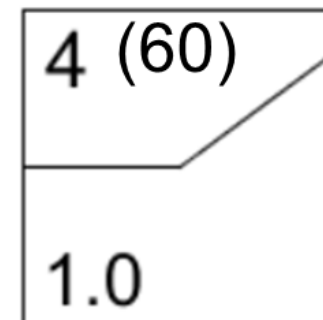

Supplement: Supplementary file 7 — Additional file 7: Profiles order based on the P-value significance of number assigned versus expected. Numbers in the backets indicate the number of the DEGs assigned. (PDF 206 KB) [file 12864_2014_6499_MOESM7_ESM.pdf]

# PLANT HORMONE SIGNAL TRANSDUCTION

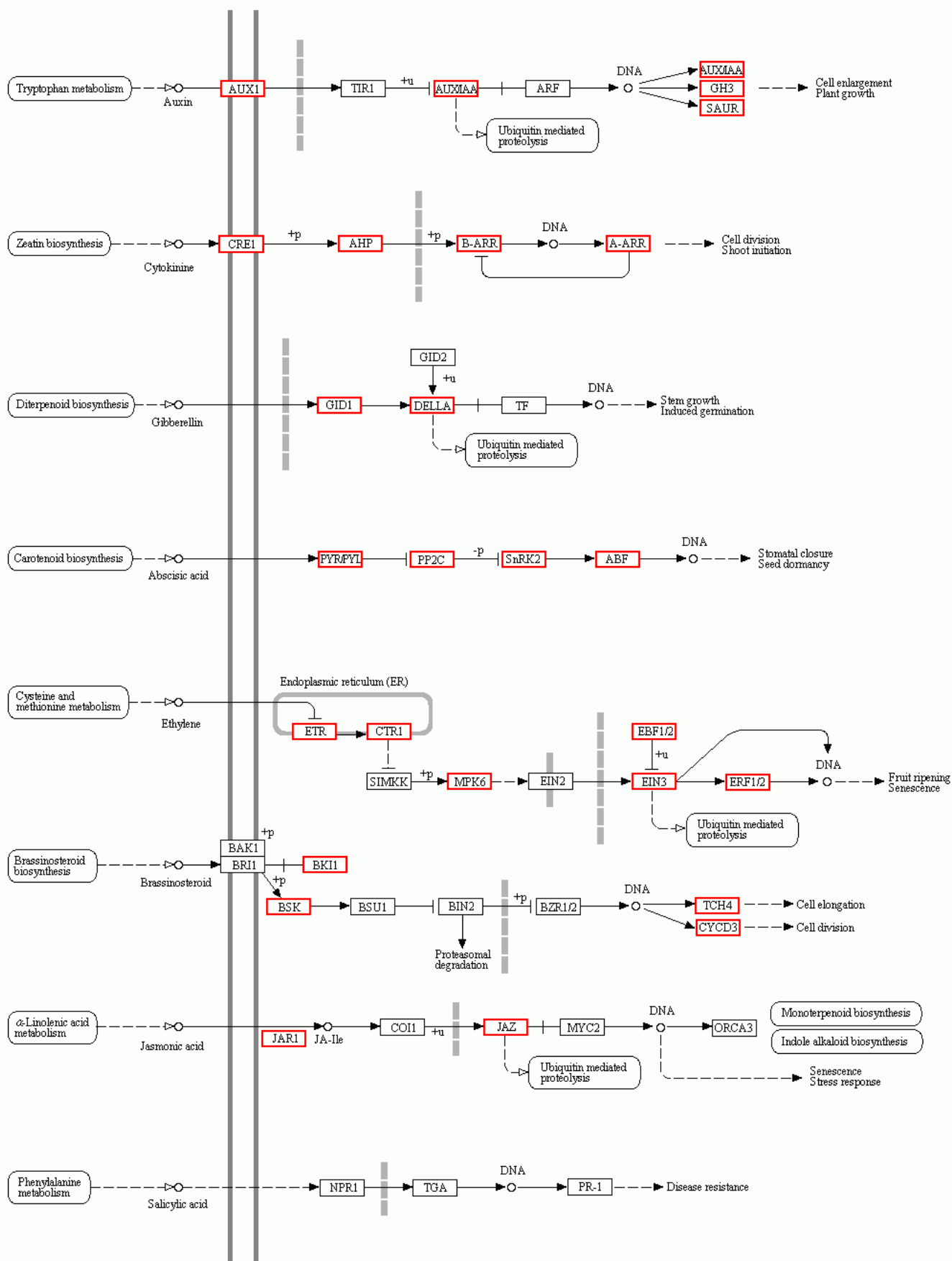

Supplement: Supplementary file 9 — Additional file 9: Enriched plant hormone signal transduction pathway. The signal transduction components marked with red rectangles are considered to be differentially expressed. (PDF 98 KB) [file 12864_2014_6499_MOESM9_ESM.pdf]
