# Supplementary material for: De novo transcriptome assembly for rudimentary leaves in Litchi chinesis Sonn. and identification of differentially expressed genes in response to reactive oxygen species
Source: BMC Genomics. 2014 Sep 20;15(1):805. doi: 10.1186/1471-2164-15-805 (PMC4190417; doi:10.1186/1471-2164-15-805)
Supplement: Supplementary file 8 — Additional file 8: Primer sequences of the reference gene and candidate unigenes for qRT-PCR. (PDF 48 KB) [file 12864_2014_6499_MOESM8_ESM.pdf]

Primer sequences of the reference gene and candidate unigenes for qRT-PCR

Homology gene	Unigene ID	Forward primer (5'→3')	Reverse primer (5'→3')
Actin		AGTTTGGTTGATGTGGGAGAC	TGGCTGAACCCGAGATGAT
Class I Beta-1,3-glucanase	LC0049290	AATGTTGGAGCCTCTTAGTGC	TGCTATTGCTTGGTCTCTTCAT
Citinase	LC0046650	GCTGTCTTGAAGGCAATAACTG	ACCTGGAACTACAACTATGGAG
Beta xylosidase	LC0042331	CCAGGATAGCCAACCCAC	TTGACCAGTCCATAGAAGCAGA
polygalacturonase	LC0036733	TTTCTGGGCTGGCGAGTT	AAGCATACCTGACCCGATAC
Phenylalanine ammonia-lyase	LC0032641	ACAAGTTCAGCAAGATACGA	AGTGTGACAAGGTGTTTACGGC
Peroxidase precursor	LC0017420	ATCCAACCTTAGATGCCACA	AGTTGTTATCAAATCCGTTAGG
cellulose synthase	LC0047175	AATAGCCTGTCCTCCAGCCTCT	CACGAACCTGTCAAACTGCCTT
Abscisic acid receptor PYL5-like	LC0076273	CAATCTGAGCCAGGGACT	GCTTCAGCGTAGTTGGTG
Abscisic acid receptor PYR1	LC0059406	GTCGTCGTCCAGAATGTC	TACCAACTCCACTCAAACCAAT
Protein phosphatase 2C (PP2C)	LC0032232	ACAACAGAAACGGCAGAA	GAATAGCACTTTGGACGC
Serine/threonine-protein kinase SRK2E-like isoform 1	LC0030437	TATCGCTGAAGCCACCAT	ACACCCCAGTAATCCAAG
Abscisic acid responsive element-binding protein 2	LC0035532	GTCTCCTCAGCAGTCCA	GCCTTCAGTCTATTCGTTG
Ethylene response 3	LC0020885	TGATTCAGTTCGTGGCATT	CCATTTCAGGAGCAGAGG
Mitogen-activated protein kinase	LC0022597	TCAACGGCGGAAGTCAAA	TCAACACCGAGCAAACGA
Ein3-binding f-box protein 3	LC0036588	AAGTATTCCAGAGCGACA	CTCAGCAACAAAACTATCC
Ethylene-insensitive 3b	LC0035971	AGGAATGAGGGTTGTAAT	TCTTGTAAGGCGGAGGAC
ERF transcription factor 4	LC0002478	GCTCTATCATAAGCCAAGG	AAGAAGCCAAACCAACAC
